# Assessing Feasibility and Acceptability of Web-Based Enhanced Relapse Prevention for Bipolar Disorder (ERPonline): A Randomized Controlled Trial

**DOI:** 10.2196/jmir.7008

**Published:** 2017-03-24

**Authors:** Fiona Lobban, Alyson L Dodd, Adam P Sawczuk, Ozgur Asar, Dave Dagnan, Peter J Diggle, Martin Griffiths, Mahsa Honary, Dawn Knowles, Rita Long, Richard Morriss, Rob Parker, Steven Jones

**Affiliations:** ^1^ Spectrum Centre Faculty of Health and Medicine Lancaster University Lancaster United Kingdom; ^2^ Department of Psychology Northumbria University Newcastle Upon Tyne United Kingdom; ^3^ Institute of Psychology Health and Society University of Liverpool Liverpool United Kingdom; ^4^ Department of Bio-statistics and Medical Informatics Acibadem University Istanbul Turkey; ^5^ CHICAS Faculty of Health and Medicine Lancaster University Lancaster United Kingdom; ^6^ Cumbria Partnership NHS Foundation Trust Penrith Cumbria United Kingdom; ^7^ Department of Psychiatry and Applied Psychology Institute of Mental Health University of Nottingham Nottingham United Kingdom

**Keywords:** Internet, randomized controlled trial, feasibility studies, bipolar disorder

## Abstract

**Background:**

Interventions that teach people with bipolar disorder (BD) to recognize and respond to early warning signs (EWS) of relapse are recommended but implementation in clinical practice is poor.

**Objectives:**

The objective of this study was to test the feasibility and acceptability of a randomized controlled trial (RCT) to evaluate a Web-based enhanced relapse prevention intervention (ERPonline) and to report preliminary evidence of effectiveness.

**Methods:**

A single-blind, parallel, primarily online RCT (n=96) over 48 weeks comparing ERPonline plus usual treatment with “waitlist (WL) control” plus usual treatment for people with BD recruited through National Health Services (NHSs), voluntary organizations, and media. Randomization was independent, minimized on number of previous episodes (<8, 8-20, 21+). Primary outcomes were recruitment and retention rates, levels of intervention use, adverse events, and participant feedback. Process and clinical outcomes were assessed by telephone and Web and compared using linear models with intention-to-treat analysis.

**Results:**

A total of 280 people registered interest online, from which 96 met inclusion criteria, consented, and were randomized (49 to WL, 47 to ERPonline) over 17 months, with 80% retention in telephone and online follow-up at all time points, except at week 48 (76%). Acceptability was high for both ERPonline and trial methods. ERPonline cost approximately £19,340 to create, and £2176 per year to host and maintain the site. Qualitative data highlighted the importance of the relationship that the users have with Web-based interventions. Differences between the group means suggested that access to ERPonline was associated with: a more positive model of BD at 24 weeks (10.70, 95% CI 0.90 to 20.5) and 48 weeks (13.1, 95% CI 2.44 to 23.93); increased monitoring of EWS of depression at 48 weeks (−1.39, 95% CI −2.61 to −0.163) and of hypomania at 24 weeks (−1.72, 95% CI −2.98 to −0.47) and 48 weeks (−1.61, 95% CI −2.92 to −0.30), compared with WL. There was no evidence of impact of ERPonline on clinical outcomes or medication adherence, but relapse rates across both arms were low (15%) and the sample remained high functioning throughout. One person died by suicide before randomization and 5 people in ERPonline and 6 in WL reported ideas of suicide or self-harm. None were deemed study related by an independent Trial Steering Committee (TSC).

**Conclusions:**

ERPonline offers a cheap accessible option for people seeking ongoing support following successful treatment. However, given high functioning and low relapse rates in this study, testing clinical effectiveness for this population would require very large sample sizes. Building in human support to use ERPonline should be considered.

**Trial registration:**

International Standard Randomized Controlled Trial Number (ISRCTN): 56908625; http://www.isrctn.com/ISRCTN56908625 (Archived by WebCite at http://www.webcitation.org/6of1ON2S0)

## Introduction

### Bipolar Disorder

Bipolar disorder (BD) is a lifelong mental health condition characterized by extreme fluctuating mood including recurrent episodes of depression and mania, which generally starts in adolescence and affects approximately 1-1.5% of adults worldwide [1]. The impact of BD on employment and relationships can be devastating, and the condition has high financial costs, estimated at £5.2 billion annually in England alone [2]. Preventing relapse is a key goal of most interventions for BD. Interventions that teach people to recognize and respond to early warning signs (EWS) are recommended by clinical guidelines worldwide [3-5] but implementation in routine clinical practice is poor [6]. Enhanced Relapse Prevention (ERP), a structured manualized intervention for frontline care staff, has shown significant benefit and is well received by patients and staff [7]. However, delivered face-to-face it will only ever be available to a small percentage of people with BD due to low rates of psychological intervention provision even among those who remain in secondary care services. In this study, we test the feasibility and acceptability of a Web-based version of ERP: ERPonline. Web-based interventions in mental health offer the potential to broaden access, reduce waiting times, delivery costs and stigma, and improve quality through standardized delivery [8,9]. There is growing evidence for short-term benefits of Internet-delivered psychological treatments for depression and anxiety disorders compared with waitlist (WL) controls [10], although understanding their implementation into real-world services is still in its infancy [11]. In BD, the evidence, while promising, is at an earlier stage, comprising small-scale feasibility studies [12-18]. These studies, along with results from an international multisite survey [19], suggest that people with BD can use, and are interested in further using, Web-based mental health support. However, detailed evidence is lacking on what kinds of psychosocial support can be Web-based, the best ways to deliver these, who accesses Web-based interventions, what processes and outcomes are impacted on, and how to best design rigorous trials to evaluate them on the Web. This information is essential to inform definitive clinical and cost-effectiveness trials. This study addresses these issues in a novel randomized controlled trial (RCT) to assess feasibility and acceptability of ERPonline with all recruitment and assessments of outcome performed remotely.

### Objectives

The objectives of this study were as follows:

First, to assess the feasibility of (1) creating a Web-based version of enhanced relapse prevention for BD (ERPonline) and (2) an RCT design using Web-based and telephone data collection to evaluate effectiveness.

Second, to determine the acceptability of ERPonline for people with BD via (1) ERPonline website usage, (2) number and type of adverse events associated with site use, and (3) detailed feedback from participants about their experiences of ERPonline to inform future developments.

Third, to determine the feasibility and acceptability of data collection via the Internet and telephone measured by recruitment and retention rates, data completion, and direct feedback from participants.

Fourth, to test the impact of the intervention on hypothesized mechanisms of change to understand processes underlying any impact.

Finally, to estimate the likely effect size of the intervention on a range of outcomes, particularly noting any negative impacts.

## Methods

### Design

A single-blind RCT with nested qualitative study comparing ERPonline plus usual treatment with a “waitlist control” arm with delayed access to ERPonline plus usual treatment. Primary outcomes were feasibility and acceptability. Process and clinical outcomes were assessed to identify measures sensitive to change collected remotely and to explore potential positive and negative impacts of the intervention. Remote data collection and online recruitment increased the external validity of the trial by encouraging participation from those unable or unwilling to engage in face-to-face clinical trials, who are also more likely to be those people unable or unwilling to engage in face-to-face clinical support for whatever reason. The study was not powered to test statistically significant impact. The trial was preregistered and full protocol published [16]. Ethics approval was given by UK National Research Ethics Service (NRES) Committee North West (Ref 12/NW/0594).

### Participants

We aimed to recruit 125 participants, anticipating a dropout rate of up to 35% (based on retention rates from previous trials of Web-based interventions for BD [12-18]) providing 40 people per arm, sufficient to meet the aims of our study to assess feasibility and acceptability. Participants were aged ≥18 years, residents of the United Kingdom, with a confirmed diagnosis of BD (1 or 2), at risk of relapse (≥3 previous episodes, ≥1 in the preceding 2 years), and with access to the Internet. We excluded people in current episode (within previous 4 weeks), currently under Mental Health Act section and therefore likely to be in current episode or at high risk of harm to self or others, or unable to understand English sufficiently to engage with the study.

### Recruitment Strategy

The study was presented to clinical teams in 8 NHS Mental Health Trusts in England, and staff were reminded in monthly team meetings to direct service users to the Web-based registration site. An advert was placed in a UK charity newsletter (Bipolar UK), and on a charity website (Bipolar Scotland). A link to ERPonline was put in NHS Choices, and British Broadcasting Corporation (BBC) health online presented a short article that linked to the website. The research team regularly tweeted about the study, and our service user lead was interviewed on local radio about the study.

People were invited to visit the site which explained the study, allowed them to check eligibility, and to register an interest in participating. Participants read online participant information sheet and completed an online consent form. Consent and capacity were reassessed at each assessment point.

### Intervention

ERPonline was developed with extensive input from a reference group of 8 adults with BD to adapt the original ERP manual to a Web-based format. Input (online and face-to-face) occurred throughout the study, but was more extensive during the initial development of the ERPonline site and included feedback on content of draft modules, user testing of the ERPonline website, and providing video and case material of lived experience which are integral parts of the intervention site. The aim of the intervention is to help people develop a coherent working model of their mood changes, recognize and manage triggers and EWS, and develop coping strategies to manage these effectively. Key modules are summarized in Table 1 with more detail in the protocol paper [20].

**Table 1:** Key intervention modules in Web-based enhanced relapse prevention intervention (ERPonline).

Section	Module title	Module description	ERPonline (n=47) average number of module views per person	ERPonline (n=47) average time spent per module per person (min)
			Mean (SD^a^) Median^b^ (min-max)	Mean (SD^a^) Median^b^ (min-max)
Getting started	How to use the site	Ways to navigate the site to get the best from the available modules	7.43 (5.77) 7 (0-25)	8.61 (8.70) 7.5 (0-46.5)
	Introduction	Explains what ERPonline is, rationale for this approach, why it might be useful, and how to involve a relative or friend if desired	4.28 (5.46) 3 (0-30)	5.88 (8.99) 2 (0-40)
	What is bipolar?	Background information about what bipolar disorder is, theories about causes, common consequences, and an overview of available treatments	8.00 (7.34) 7 (0-30)	11.95 (13.51) 7 (0-52.5)
Key Modules	Mood charting	How to use a Web-based tool to monitor mood on a daily basis to help recognize normal mood fluctuation and pick up early signs of a mood episode	138.38(445.54) 13 (0-2519)	122.89 (386.43) 14.5 (0-2150.5)
	Life charting	Complete a chart of past mood episodes, identifying potential triggers and coping strategies for future mood changes	49.09 (147.55) 11 (0-990)	47.34 (114.45) 8.00 (0-744)
	Identifying triggers	Detailed analysis of triggers of previous mood episodes, followed by a personalized plan of how to manage triggers	11.34 (25.57) 1 (0-148)	15.07 (30.09) 0.5 (0-140.5)
Specific moods	Early warning signs (EWS) -high mood	Detailed analysis of EWS of high mood to develop a relapse signature for (hypo) mania	14.47 (26.72) 0 (0-90)	17.36 (32.31) 0 (0-114)
	Coping strategies -high mood	Review of current strategies to manage high mood and introduction to new strategies that may be helpful	5.68 (10.62) 0 (0-44)	10.0 (19.78) 0 (0-71.5)
	Early warning signs (EWS) -low mood	Detailed analysis of EWS of low mood to develop a relapse signature for depression	9.26 (22.47) 0 (0-94)	9.38 (21.87) 0 (0-84.5)
	Coping strategies -low mood	Review of current strategies to manage low mood and introduction to new strategies that may be helpful	3.83 (11.26) 0 (0-59)	3.95 (11.23) 0 (0-52.5)
Wrapping things up	Staying well strategies	Identifying and managing stress levels Understanding the importance of social rhythms and how to regulate these to manage mood How relationships with other people impact on mood	4.06 (6.11) 0 (0-25)	4.39 (8.30) 0 (0-37)
	Your staying well plan	An individualized summary of staying well strategies, early warning signs to look out for, and coping strategies to regulate mood	3.09 (5.94) 0 (0-30)	2.35 (4.60) 0 (0-18.5)

^a^SD: standard deviation.

^b^The median value 0 indicates that at least half the sample did not visit this module.

**Figure 1:**
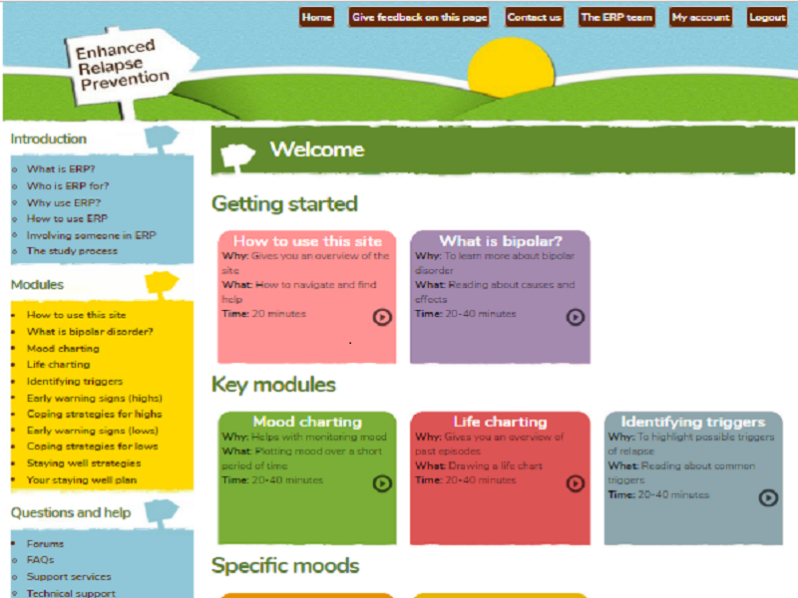
The online enhanced relapse prevention intervention (ERPonline) home page - top of page.

**Figure 2:**
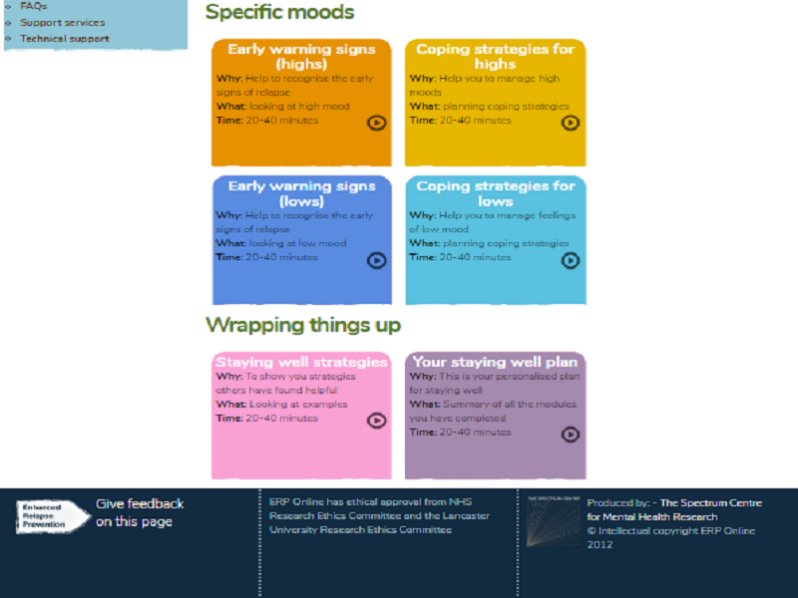
The online enhanced relapse prevention intervention (ERPonline) home page - bottom of the page.

Each module included information, suggested strategies, and case examples. Users interacted with the site to input personal information relevant to their own triggers, EWS, and coping strategies. These informed an individualized staying well plan. The site also provided signposting to additional formal and informal support. Participants were free to choose the order they visited modules (although they were listed in logical order), and were invited to involve a supporter of their choosing. Each module included recommendations of how the supporter could be involved in relapse prevention. All participants continued to receive any other treatment as usual throughout the study. The home page is shown in Figures 1 and 2 for illustration.

### Procedure: Randomization and Masking

Diagnostic eligibility was confirmed using Structured Clinical Interview for Diagnostic and Statistical Manual of Mental Disorders-IV (DSM-IV) [21] administered by telephone by trained research assistants. Training consisted of scoring training videos, conducting clinical sensitive interviews on the telephone, and recording an interview with someone with BD who provided experiential feedback and which was then rated by a supervising clinical academic. Training continued until ratings were reliable and clinical style was of high quality. Monthly supervision to ensure reliability in scoring of telephone interviews continued throughout the study. Following baseline assessments (telephone and Web-based) participants were randomly allocated by an independent clinical trials unit (CTU) using 1:1 ratio, minimized on number of previous episodes (<8, 8-20, 21+) and including a random element to minimize predictability of allocation. Those in the ERPonline arm received an email containing a weblink and instructions of how to log-on to the site using a unique username and password. Control participants received an email or telephone call informing them of the allocation and emphasizing the importance of continued participation throughout the trial. All communication with CTU and participants regarding randomization was conducted by the trial manager (unblinded). All communication with participants reiterated the importance of not telling the researcher carrying out the follow-up interviews which group they were in, and why this was important. All assessments were conducted by blind researchers. Blindness was further maintained using restricted file access to any data showing randomization, and prefacing each follow-up interview with a reminder about why it was important not to say anything about which arm they were in.

During the trial, participants were sent an additional email inviting them to provide qualitative feedback. The ERPonline group was asked to complete a Web-based survey about their views of the ERPonline site and any improvements they would recommend. They were also given the option (between 2 and 12 months following randomization) to take part in a telephone interview about their experiences of using ERPonline. Given the relatively novel primarily Web-based trial design, the WL control group was sent a survey before accessing the site about their reasons for engaging in the trial, and their experience of taking part in the trial.

A reflective log detailed our experiences throughout the trial.

### Measures

Proposed mechanisms of change, were assessed at baseline, 24 and 48 weeks including frequency of EWS monitoring (EWS checklist for relapse in depression and mania [22] Likert scale 1=never to 4=very regularly), adapted Brief Illness Perception Questionnaire (BIPQ score 0-110: higher score=more negative beliefs) [23], and the Medication Adherence Rating Scale (MARS 0-10: higher score=higher compliance) [24]. These measures were either designed for use with people with BD (EWS checklists and BIPQ), or have been successfully used with this population (MARS). They are all self-report, have high face validity, and have been shown to be valid and reliable measures, making them highly applicable to Web-based use.

Interviewer-rated outcome measures were administered by telephone by two trained research assistants, at baseline, 12-, 24-, 36-, and 48-week follow-up. These included SCID-LIFE [25] providing a retrospective weekly rating of depression (1-6) and mania (1-6) (scores of 5 or 6 indicate major mood episode); Hamilton Depression Rating Scale (HAM-D scores above 7 indicate mild depression, above 13 moderate, and above 18 severe) [26], and Mania Rating Scale (MRS scores of 11 and above indicate hypomania) [27]; the Personal and Social Performance Scale (PSP scores 70-80 indicate mild difficulties, above 80 is good functioning) [28]; and the Multidimensional Scale of Independent Functioning (MSIF Likert scale 1=normal functioning, to 7=total disability) [29]. Self-report outcome measures were collected at baseline, 24, and 48 weeks and included the Work and Social Adjustment Scale (WSAS less than 10=subclinical; 10-20 some functional impairment; above 20 moderate psychopathology) [30]; Quality of Life in Bipolar Disorder (QoLBD range 48-240 with high score=higher quality of life) [31]; and the Bipolar Recovery Questionnaire (BRQ score 0-3600, high score=higher recovery) [32]. Web-based versions of the EQ5D5L [33] and the Client Service Receipt Inventory (CSRI) [34] were piloted to assess the feasibility of collecting this data on the Web and to test the sensitivity to change in this population as neither have been previously used in this format. A checklist to record current treatment was developed for the study to define usual treatment.

The only change to the published protocol was to record only the frequency of monitoring of EWS for hypomania and depression, as early feedback from participants indicated the full checklist was too long. All serious adverse events (SAEs) were recorded and reported to the Trial Steering Committee (TSC). All participants were given a £10 shopping voucher on completion of measures at each assessment point.

### Analysis

Descriptive statistics report the characteristics of the sample recruited; use of the website; and rates of recruitment, retention, and data completion in each arm of the trial. The impact on repeated process and outcome measures was tested using linear models with correlated errors, which allow for correlation between repeated measures from the same participant. For ordinal data, we used generalized linear mixed models. We report both unadjusted analyses, and those adjusting for any differences in baseline demographic (age, gender, ethnicity, employment, education) and clinical variables (number of previous episodes, and whether or not prescribed a mood stabilizer).

Incomplete records from participants were retained, and analyses used maximum likelihood estimation for all model parameters. Statistical comparison of outcomes was made between the two trial arms at 24 and 48 weeks follow-up.

Weekly ratings of depression and mania from the SCID-LIFE were used to analyze time to first relapse (any and separately for depression [requiring 2 consecutive weeks], or mania [1 week]) and the proportion of time spent in episode (defined as SCID-LIFE rating of 5 or 6), or in subsyndromal state (SCID-LIFE rating of 3 or 4) or euthymic (SCID-LIFE rating of 1 or 2). To analyze the impact of the intervention on time to first relapse, we used a Cox’s proportional hazards regression model. Beta regression was used to compare the proportion of time spent in episode or subsyndromal or euthymic in each arm.

The study is not powered to test for statistically significant impact and therefore we do not specify a primary outcome, or set a level of statistical significance for interpreting analyses. All analyses were run from R open-source computing environment version 3.3.1 (R Foundation for Statistical Computing).

Content analysis of qualitative survey data highlighted the individual points made and these were grouped into key themes. Interview transcripts were analyzed in depth using indexing and charting methods inspired by Framework Analysis [35]. All transcripts were independently coded by the interviewer (MG) and a second member of the research team. Codes were compiled into a tentative coding frame with thematic headings. Narrative summaries were created from each of the conceptual themes across all cases. This data will be reported in full elsewhere but here we present key data relevant to the feasibility and acceptability aims of the trial.

## Results

### Quality Assurance

Only two unblindings occurred. In both instances, the participant inadvertently indicated their group during a telephone assessment (one WL control, one ERPonline). Subsequent assessments were completed by a blind Research assistant. At each follow-up, 10% of the SCID-LIFE interviews were rated by both researchers and kappa statistic calculated to assess interrater reliability. These ranged from acceptable (κ=.54, 95% CI 0.39-0.69, at 36 weeks based on 142 weekly ratings for 6 participants) to high (κ=.90, 95% CI 0.82-0.98, at 12 weeks based on 192 weekly ratings for 8 people).

### Feasibility and Acceptability of Trial Design

**Figure 3:**
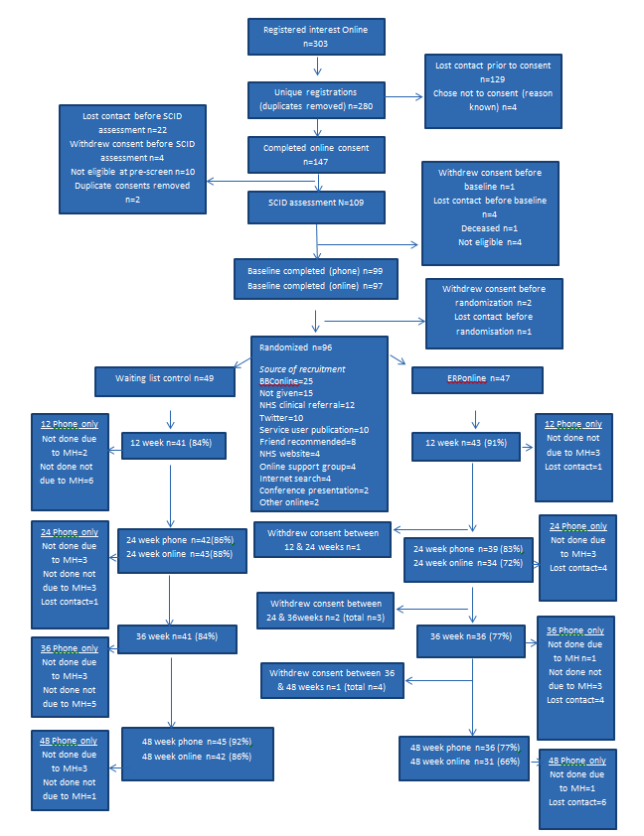
The online enhanced relapse prevention intervention (ERPonline) consort diagram.

Participant flow is detailed in Figure 3, including recruitment, over half of which came via online sources. A total of 96 people were randomized (49 to WL, 47 to ERPonline) over a 17-month period, with 80% retention in telephone and online follow-up at all time points, except week 48 (76%). Attrition was 11% lower in WL arm.

Participants were predominantly diagnosed with BD1 (90%) and had a chronic relapsing course (67% had over 21 relapses; see Table 2). Despite this, the group was currently high functioning and had a positive attitude to recovery. The vast majority were taking and adherent to medication to manage their mood and over half had previously received psychological treatment for BD (where specified, this was most commonly described as cognitive behavior therapy [CBT]).

**Table 2:** Key characteristics of participant sample at baseline.

Participant characteristics		Wait list (n=49)	ERPonline (n=47)
Baseline demographic and clinical variables			
**Age, mean (SD^a^** **)**		43.8 (11.45)	42 (12.23)
**Gender, n (%)**			
	Female	32 (65)	27 (57)
**Ethnicity, n (%)**			
	White British	44 (90)	38 (81)
	Any other white	2 (4)	5 (11)
	Black British	-	1 (2)
	Caribbean	1 (2)	-
	Asian British	1 (2)	-
	Indian	-	1 (2)
	Any other mixed	-	1 (2)
	Missing	1 (2)	1 (2)
**Occupational status, n (%)**			
	Full-time paid or self	16 (33)	21 (45)
	Part-time paid or self	13 (27)	6 (13)
	Voluntary	3 (6)	4 (9)
	Not employed	10 (20)	6 (13)
	Student	3 (6)	2 (4)
	Housewife or househusband	-	3 (6)
	Retired	4 (8)	5 (11)
**Education, n (%)**			
	No formal qualifications	1 (2)	-
	CSE^b^ or O Level or GCSE^c^	5 (10)	4 (9)
	A Level	7 (14)	7 (15)
	Degree	17 (35)	16 (34)
	PG^d^ Diploma or qualification	13 (27)	13 (28)
	Doctorate or PhD	3 (6)	7 (15)
**Total number of past episodes (baseline), n (%)**			
	<7	6 (12)	2 (4.)
	8-20	12 (25)	12 (26)
	21+	31 (63)	33 (70)
**Taking mood stabilizer (baseline), n (%)**			
	Not on any medication (so item rated not applicable)	3 (6)	2 (4)
	No mood stabilizer	15 (31)	11 (23)
	Lithium	12 (25)	11 (23)
	Sodium valproate	5 (10)	14 (30)
	Carbamazepine	3 (6)	1 (2)
	Lamotrigine	11 (22)	8 (17)
**Treatment history (baseline), n (%)**			
	Ever used mental health services	46 (94)	42 (89)
	Clinical diagnosis of BD1^e^ (vs BD2)	44 (90)	44 (94)
	Ever seen a psychiatrist	46 (94)	43 (92)
	Ever prescribed medication for BD	49 (100)	46 (98)
	Currently taking medication for bipolar disorder	40 (82)	40 (85)
	Ever received therapy or psychosocial intervention for bipolar disorder	26 (53)	27 (57)
	Currently receiving therapy for bipolar disorder	8 (16)	10 (21)
Process measures			
**Early warning signs monitoring frequency—** **depression, n (%)**			
	Never	3 (6)	3 (6)
	Occasionally	18 (37)	11 (23)
	Fairly regularly	8 (37)	20 (43)
	Very regularly	10 (20)	13 (28)
**Early warning signs monitoring frequency—** **hypomania, n (%)**			
	Never	6 (12)	5 (11)
	Occasionally	21 (43)	14 (30)
	Fairly regularly	16 (33)	18 (38)
	Very regularly	6 (12)	10 (21)
**Brief Illness Perception Questionnaire—total (high score=more negative model), mean (SD)**		60.6 (10.5)	60.7 (9.9)
**Medication Adherence Rating Scale, mean (SD)**		6.9 (2.2)	7.0 (2.1)
Outcome measures			
**Hamilton Depression Rating Scale, mean (SD)**		4.5 (5.3)	3.5 (4.2)
**Mania Rating Scale, mean (SD)**		1.3 (2.4)	1.0 (1.7)
**Personal and Social Performance Scale, mean (SD)**		79.7 (11.2)	79.1 (13.1)
**Multidimensional Scale of Independent Functioning, n (%)**		25 (51) 14 (29) 8 (16) 2 (4) 0 (0) 0 (0)	21 (45) 16 (34) 6 (13) 2 (4) 2 (4) 0 (0)
**Work and Social Adjustment Scale, mean (SD)**		11.4 (9.4)	12.8 (8.7)
**Quality of Life in Bipolar Disorder, mean (SD)**		162.7 (33.5)	162.5 (22.8)
**Bipolar Recovery Questionnaire, mean (SD)**		2332 (394)	2342 (383)

^a^SD: standard deviation.

^b^CSE: Certificate of Secondary Education.

^c^GCSE: General Certificate of Secondary Education.

^d^PG: postgraduate.

^e^BD: bipolar disorder.

### Feasibility and Acceptability of Data Collection

Telephone and Web-based data collection procedures were generally acceptable to participants based on information from 41 participants (WL survey, n=22; ERPonline interviews, n=19 reported separately). Survey data indicated factors that encouraged people to take part including opportunity to improve their own resilience and self-management, wanting to help others, and recognizing the importance of research on improving Web-based interventions, due to a perceived gap in face-to-face services and some existing websites feeling unsafe. Many of these factors were also cited as facilitating retention in the trial, as well as factors such as text reminders about follow-ups, and viewing the research team as sensitive, polite, and nonintrusive. Participants reported that the research process was well-managed, clearly explained, straightforward, and flexible, and they liked the shopping vouchers. Some participants believed completing the measures had changed their thinking about their mood and diagnosis. Barriers to retention included procedural difficulties, such as remembering follow-up times and rescheduling missed appointments, feeling “weird” to have interviews only on the phone and difficulties finding private space for the phone calls; issues with measures, some of which were too long (CSRI) and could be tiring, distressing, and required recall over long periods of time; and technical difficulties with Web-based questionnaires. Some reported feeling disappointed to be in the WL control arm, although had remained in the trial.

Additional key data collection lessons we learnt included the importance of checking electronic communication is received (some of our reminder emails were initially going into junk folders), the need to accommodate the high demand for evening and weekend telephone appointments, and the importance of text reminders for telephone appointments.

### Feasibility and Acceptability of ERPonline Intervention

ERPonline is low cost at an estimated £19,340 to create, and approximately £2176 per year to host and maintain the site. Development costs included time to adapt content from the ERP manual; discussion and feedback with coauthors; Web developer time to build the site; filming and producing videos; and costs for the Service User Reference Group to feedback on early iterations. Hosting costs include software updates and technical issues (estimated at 2 h per week) and space on a server. To keep costs low, ERPonline was delivered unsupported and without a Web-based moderated forum, despite these being part of the intended design.

Activity levels were highly skewed. Two people allocated to ERPonline never visited the site. Mean number of page views per person was 259 (SD 577), median was 85 (range 0-3203). Participants spent a mean of 259 min (SD 509), median 76 min (range 0-2770) accessing ERPonline throughout the 48-week intervention period. The most frequently viewed modules were “Life Charting” (median views 11; range 0-990 per person) and “Mood Charting” (median views 13; range 1-2519 per person), which is unsurprising as they offered an ongoing monitoring function. “Coping strategies for Low Mood” (median views 0: range 0-44 per person) and “Your Staying Well Plan” (median views 0; range 0-30 per person) were the least frequently visited, but also occurred toward the end of the listed modules (see Table 1 for number of visits and time spent on all modules).

A total of 17 ERPonline participants (36%) responded to the Web-based survey. Overall these participants were satisfied (13/17 people [76%] somewhat or very satisfied), found it somewhat or very helpful (12/17, 71%), and very or extremely relevant (13/17, 76%). Only one person said they would not recommend it to a friend. Most useful features were recognizing EWS of relapse, shared experiences through videos, mood monitoring, ability to revisit and refresh skills, improved knowledge and self-management of BD, ease of use, and being able to use the site with the family. The key recommendation for improvement was additional support with working through the materials. The sample of questionnaire respondents described themselves as confident (n=16, 94% very or extremely confident) and regular (n=12, 71% at least daily) Internet users.

A total of 19 people took part in qualitative interviews about their experience of the trial and use of the ERPonline site. The key finding was the importance of relationships that the individual developed with the ERPonline team in determining retention into the study and use of the site:

I think the sort of general thoroughness and kindness of the people I dealt with that certainly contributed to, you know, me sort of staying in the study. Everybody’s been really upbeat, very positive, very accommodating

P10

This is particularly interesting when we consider there was no face-to-face contact. Sole direct contact was by telephone at three monthly interviews for SCID-LIFE interviews, which for some was preferable to face-to-face:

…I think it being over the ‘phone makes it a bit easier. If it’s face-to-face I would have probably not been quite so comfortable answering. But yeah over the ‘phone was definitely not so bad.

P13

Crucial to the strength of the alliance was the perceived trustworthiness of the team and being made aware of the extensive user involvement in design and content of the site:

It’s always available and also the information’s on there has been put together by the people who do know what they’re doing.

P7

I suppose the prospect of the online study kept me quite interested and the fact that it was developed by other people with Bipolar and that was something that I was definitely interested in…

P17

A key recommendation to improve the ERPonline site was to integrate human support to facilitate ongoing use of the site. This is consistent with previous studies in which adherence was higher in groups receiving a Web intervention plus support as compared with Web intervention only [31]. Participants felt websites should be used to support interventions delivered by real people and not as a cheaper replacement:

The cash strapped health service will rely heavily on these sort of techniques which I think only fill one part of the market. I think they only really deal with, you know, and a comparatively narrow field of potential patients. I think they’re very useful but I do think the gold standard involves some sort of face-to-face psychological therapy. And I think the clinical literature bears that out so, I want more jobs for psychologists basically

Henry: 23.893-23.898

**Table 3:** Descriptive statistics on outcome and process measures at 24- and 48-week follow-ups.

Variable	Group	Baseline, n WL^a^=49(I^c^); 49(O^d^) ERP^b^=47(I); 47(O)	12 weeks WL=41(I) ERP=43 (I)	24 weeks WL=42(I); 43(O) ERP=39(I); 34(O)	36 weeks WL=41(I) ERP=36(I)	48 weeks WL=45(I); 42(O) ERP=36(I); 31(O)
**Process measures**						
Early warning signs monitoring frequency—depression (O), n (%)	never WL	3 (6)		8 (19)		4 (10)
	Occasionally	18 (37)		13 (30)		11 (26)
	Fairly regularly	18 (37)		17 (40)		19 (45)
	Very regularly	10 (20)		5 (12)		6 (14)
	never ERP	3 (6)		1 (3)		1 (3)
	Occasionally	11 (23)		11 (32)		6 (19)
	Fairly regularly	20 (43)		15 (44)		10 (32)
	Very regularly	13 (28)		7 (21)		14 (45)
Early warning signs monitoring frequency—hypomania (O), n (%)	never WL	6 (12)		11 (26)		4 (10)
	Occasionally	21 (43)		17 (40)		18 (43)
	Fairly regularly	16 (33)		9 (21)		11 (26)
	Very regularly	6 (12)		6 (14)		7 (17)
	never ERP	5 (11)		2 (6)		1 (3)
	Occasionally	14 (30)		10 (29)		8 (26)
	Fairly regularly	18 (38)		15 (44)		11 (35)
	Very regularly	10 (21)		7 (21)		11 (35)
BIPQ^e^—total (O), mean (SD^f^)	WL	60.6 (10.5)		49.4 (22.6)		49.4 (24.6)
	ERP	60.7 (9.9)		39.3 (27.0)		36.2 (29.1)
MARS^g^ (O), mean (SD)	WL	6.9 (2.2)		6.7 (2.6)		6.6 (2.5)
	ERP	7.0 (2.1)		6.8 (2.7)		7.0 (2.2)
**Outcome measures**						
HAM-D^h^ (I), mean (SD)	WL	4.5 (5.3)	7.5 (7.4)	7.3 (8.6)	6.8 (8.6)	8.2 (9.0)
	ERP	3.5 (4.2)	6.6 (6.7)	6.9 (8.0)	6.0 (8.3)	7.1 (9.3)
MRS^i^ (I), mean (SD)	WL	1.3 (2.4)	2.7 (3.7)	2.2 (4.1)	1.7 (2.9)	1.7 (2.2)
	ERP	1.0 (1.7)	2.5 (4.4)	2.4 (3.9)	2.0 (4.0)	1.4 (2.4)
PSP^j^ (I), mean (SD)	WL	79.7 (11.2)	75.0 (15.1)	76 (16.4)	79.8 (14.8)	78.4 (15.6)
	ERP	79.1 (13.1)	77.8 (15.1)	76.7 (15.4)	77.8 (16.1)	80.7 (16.1)
MSIF^k^-global (frequencies for scores categories 1, 2, 3, 4, 5, 6) (I), n (%)	WL					
	1	25 (51)	24 (59)	20 (48)	25 (61)	28 (62)
	2	14 (29)	8 (20)	13 (31)	10 (24)	9 (20)
	3	8 (16)	4(10)	5 (12)	3 (7)	6 (13)
	4	2 (4)	5 (12)	3 (7)	1 (2)	1 (2)
	5	0 (0)	0 (0)	1 (2)	2 (5)	0 (0)
	6	0 (0)	0 (0)	0 (0)	0 (0)	1 (2)
	ERP					
	1	21 (45)	26 (60)	18 (46)	20 (56)	22 (61)
	2	16 (34)	10 (23)	12 (31)	9 (25)	9 (25)
	3	6 (13)	3 (7)	6 (15)	5 (14)	4 (11)
	4	2 (4)	3 (7)	2 (5)	0 (0)	1 (3)
	5	2 (4)	1 (2)	1 (3)	2 (6)	0 (0)
	6	0 (0)	0 (0)	0 (0)	0 (0)	0 (0)
WSAS^l^ (I), mean (SD)	WL	11.4 (9.4)		12.4 (10)		12.9 (10.6)
	ERP	12.8 (8.7)		14.3 (9.1)		14.8 (10.3)
QoLBD^m^ (O), mean (SD)	WL	162.7 (33.5)		161.2 (39.7)		154.9 (36.1)
	ERP	162.5 (22.8)		156.5 (33.4)		151.8 (41.7)
BRQ^n^ (O) mean (SD)	WL	2332 (394)		2309 (504)		2336 (468)
	ERP	2342 (383)		2451 (430)		2414 (577)

^a^WL: waitlist.

^b^ERP: enhanced relapse prevention.

^c^I: interviewer rated by telephone.

^d^O: completed online.

^e^BIPQ: Brief Illness Perception Questionnaire (score 0-110 higher score=more negative beliefs).

^f^SD: standard deviation.

^g^MARS: Medication Adherence Rating Scale (0-10 higher score=higher compliance).

^h^HAM-D: Hamilton Depression Rating Scale (scores above 7 indicate mild depression, above 13 moderate, and above 18 severe).

^i^MRS: Mania Rating Scale (scores of 11 and above indicate hypomania).

^j^PSP: Personal and Social Performance Scale (scores 70-80 indicate mild difficulties, above 80 is good functioning).

^k^MSIF: Multidimensional Scale of Independent Functioning (Likert scale 1=normal functioning, to 7=total disability).

^l^WSAS: Work and Social Adjustment Scale (less than 10=subclinical; 10-20 some functional impairment; above 20 moderate psychopathology).

^m^QoLBD: Quality of Life in Bipolar Disorder (range 48-240 with high score=higher quality of life).

^n^BRQ: Bipolar Recovery Questionnaire (score 0-3600 high score=higher recovery).

**Table 4:** Comparison of linear models with correlated errors to test for differences between waitlist (WL) and Web-based enhanced relapse prevention intervention (ERPonline) on outcome and process measures at 12-, 24-, and 48-week follow-ups. Unadjusted model showing estimates of difference between beta estimates at each time point.

Variable	Model 1—Unadjusted analysis								
	12-week follow-up estimate	95% CI (*P* value)	24-week follow-up estimate	95% CI (*P* value)	36-week follow-up estimate	95% CI (*P* value)	48-week follow-up estimate	95% CI (*P* value)
Early warning signs monitoring frequency—depression			0.73	−1.86 to 0.40 (.20)			−1.39	−2.61 to −.163 (.03)
Early warning signs monitoring frequency—hypomania			−1.72	−2.98 to −.47 (.01)			−1.61	−2.92 to −.30 (.02)
Brief Illness Perception Questionnaire^a^ —total			10.70	0.90 to 20.5 (.03)			13.18	2.44 to 23.93 (.02)
Medication Adherence Rating Scale			−0.102	−1.07 to 0.87 (.84)			−0.327	−1.34 to 0.685 (.53)
Personal and Social Performance Scale	−2.91	−9.19 to 3.37 (.36)	−0.198	−6.77 to 6.38 (.95)	0.77	−5.87 to 7.41 (.82)	−2.87	−9.27 to 3.52 (.38)
Multidimensional Scale of Independent Functioning—global (frequencies for scores categories 1, 2, 3, 4, 5, 6)	0.169	−0.867 to 1.20 (.75)	.029	−0.959 to 1.02 (.95)	−0.074	−1.15 to 1.00 (.89)	.281	−0.785 to 1.35 (.61)
Work and Social Adjustment Scale			−1.61	−5.67 to 1.46 (.30)			−0.53	−3.90 to 2.83 (.76)
Quality of Life in Bipolar Disorder			6.67	−5.73 to 19.1 (.29)			3.99	−9.72 to 17.7 (.57)
Bipolar Recovery Questionnaire			−52.3	−195 to 90.7 (.47)			−38.09	−208.58 to 132.41 (.66)

^a^Brief Illness Perception Questionnaire total measures how negative a model the person has—so high score=more negative model.

**Table 5:** Comparison of linear models with correlated errors to test for differences between waitlist (WL) and Web-based enhanced relapse prevention intervention (ERPonline) on outcome and process measures at 12-, 24-, and 48-week follow-ups. Adjusted for any differences in baseline demographic (age, gender, ethnicity, employment, education) and clinical variables (number of previous episodes, and whether or not prescribed a mood stabilizer).

Variable	Model 2—Adjusted analysis								
	12-week follow-up estimate	95% CI (*P* value)	24-week follow-up estimate	95% CI (*P* value)	36-week follow-up estimate	95% CI (*P* value)	48-week follow-up estimate	95% CI (*P* value)
Early warning signs monitoring frequency —depression			−0.721	−1.85 to 0.413 (.21)			−1.38	−2.61 to −0.153 (.03)
Early warning signs monitoring frequency —hypomania			−1.70	−2.96 to −0.452 (.01)			−1.60	−2.91 to −0.291 (.02)
Brief Illness Perception Questionnaire^a^ —total			11.06	1.25 to 20.9 (.03)			13.6	2.69 to 24.6 (.02)	
Medication Adherence Rating Scale			−0.128	−1.11 to 0.855 (.80)			−0 **.**356	−1.39 to 0.674 (.50)
Personal and Social Performance Scale	−2.99	−8.84 to 2.863 (.32)	−0.430	−7.02 to 6.16 (.90)	.704	−6.06 to 7.47 (.84)	−3.21	−9.69 to 3.26 (.33)
Multidimensional Scale of Independent Functioning—global (frequencies for scores categories 1, 2, 3, 4, 5, 6)	0.092	−0.907 to 1.09 (.86)	−0.075	−1.03 to 0.878 (.88)	−0.084	−1.12 to 0.95 (.87)	0.275	−0.743 to 1.29 (.60)
Work and Social Adjustment Scale			−1.46	−4.53 to 1.64 (.36)			−0.40	−3.84 to 3.03 (.82)
Quality of Life in Bipolar Disorder			6.23	−6.13 to 18.6 (.32)			3.58	−10.4 to 17.5 (.62)
Bipolar Recovery Questionnaire			−63.29	−206.47 to 79.88 (.39)			−35.84	−209.78 to 138.10 (.69)

^a^Brief Illness Perception Questionnaire total measures how negative a model the person has—so high score=more negative model.

#### Estimate of Impact on Outcome and Process Measures

Descriptive statistics on process and outcome measures at each time point are shown in Table 3. Comparison between WL and ERPonline on process and outcome measures at 12-, 24-, and 48-week follow-ups are shown in Table 4. Models adjusting for baseline demographic and clinical variables are also shown in Table 5.

#### Process Measures

ERPonline increased the frequency of monitoring early signs of mood change (EWS—depression and EWS—hypomania), evident for hypomania at 24 weeks (−1.72, 95% CI −2.98 to −0.47), and for both at 48 weeks (depression −1.39, 95% CI −2.61 to −0.163; hypomania −1.61, 95% CI −2.92 to −0.30), and improved working model of mood changes (BIPQ—high score indicates more negative model) at both 24 weeks (10.70, 95% CI 0.90-20.5) and 48 weeks follow-ups (13.18, 95% CI 2.44-23.93; Table 4). All differences are robust to adjustments in model 2 for baseline differences between the groups (Table 5). Medication adherence was high (indicated by high score on the MARS) throughout the study and did not differ between groups.

#### Mood and Functioning

Depression and hypomania were low at all time points suggesting a generally stable and euthymic group. Similarly, functioning on WSAS, PSP, and MSIF at baselines were suggestive of very mild impairment in work and social performance and remained so throughout. Time spent in euthymic, subsyndromal, and relapse mood states respectively in WL were 93% (SD 8%), 5% (SD 7%), and 3% (SD 15%), and in ERPonline: 95% (SD 8%), 4% (SD 6%), and 2% (SD 4%). There were no notable differences between the two groups in any of the outcome measures at any of the time-points.

#### Relapse

Of the 96 participants, one provided no SCID-LIFE data at follow-up. Only 15 (16%) participants experienced a relapse over the 48-week follow-up; 11 (11%) depressive and 7 (7%) mania-type. There were no notable differences between groups on time to any relapse (unadjusted hazard ratio [HR] 1.67, 95% CI 0.60-4.71), *P*=.33; time to depressive episode (HR 1.53, 95% CI 0.49-4.83), *P*=.47; time to mania type episode (HR 2.87, 95% CI 0.56-14.8), *P*=.12. Given the low level relapses, no Kaplan-Meier curves are presented.

### Adverse Events

During the trial, 1 participant completed suicide before randomization, 11 participants (11% of those randomized) reported suicidality and self-harm, and 1 made a suicide attempt (before withdrawing from the study). It was found that 6 were in the WL arm, and 5 were receiving ERPonline. None of the SAEs were deemed study related by an independent TSC.

## Discussion

### Principal Findings

ERPonline is a novel Web intervention for improving relapse prevention and providing National Institute for Health and Care Excellence (NICE) congruent information to people with BD. This study indicates that the development and evaluation of this type of approach in a rigorous RCT using telephone and Web-based assessments is both feasible and acceptable. Important lessons were learnt relevant to each of our study aims, but which also have relevance to the wider development and evaluation of remote-access approaches for other health problems.

With the help of our Service User Reference Group, we were able to develop a Web-based version of an existing ERP intervention for people with BD at a very low cost that received largely positive feedback, and led to no evident adverse events. Activity was highly skewed but over 90% of our sample visited the site more than once, which can be compared with MyRecoveryPlan [17] that reported site returns for 71% in a coached group, and only 44% in an unsupported group. On the basis of levels of use of the different modules and direct participant feedback, engagement could be enhanced by making the intervention more interactive and providing support to use it.

Recruitment, retention, and data completion strategies were largely successful. Retention was higher than demonstrated in previous Web-based trials with people who are affected with BD [14,15,17]. Key features of the trial design that facilitated this included payment for completing assessments, text and email reminders, a WL control design, and a friendly flexible research team who were willing to offer telephone appointments at times to suit participants including out-of-office hours. However, to reach the sample size required for large scale clinical and cost effectiveness trials, paying for advertising through popular websites such as Google and Facebook may be necessary [36].

Feedback about the experience of taking part in a primarily Web-based trial was mixed. Some participants reported difficulties finding a private space to take telephone calls or finding Web-based measures difficult, tiring or distressing, whereas others valued the flexibility, convenience, and felt more able to be open about the problems they had experienced than in a face-to-face interview. This suggests that trials which offer a choice of data collection options may be most effective in achieving recruitment and retention targets.

However, further work is needed to test the validity and reliability of these data collection approaches. Our data showed that while the hypothesized increase in EWS monitoring and development of more positive beliefs about mood swings did occur in those receiving ERPonline compared with WL control group, we did not see any benefit of ERPonline on any of the clinical outcome measures. This was largely due to the ceiling effect on our outcome measures. Only 16% of the total sample experienced any relapse, compared with expected levels of 50-70% [37].

This ceiling effect was consistent across all outcome measures and all assessors. Therefore, the most likely explanations are either that the method of data collection is leading to underreporting of problems, or that the participating sample reflect a different population from those taking part in more traditionally designed face-to-face clinical studies.

With regards to the first possibility, whereas we did not directly test the reliability of the data compared with a face-to-face interview, other studies have done this comparing telephone and face-to-face interview data of SCID assessments found high levels of agreement [38]. Our team have also carried out a parallel Web-based RCT which included the same Web-based and telephone assessments, delivered through researchers trained by the same methods, and which will report relapse rates of 47% which are akin to those expected from previous research data and much higher than in this study [39].

The second possible explanation can be explored by examining the characteristics of our participants. Compared with bipolar samples recruited to other face-to-face trials [37] including one evaluating clinician delivered ERP [7], and samples in other Web-based trials which all show higher relapse rates [14,15,17], our sample are more euthymic, highly educated, likely to be in employment, and have had surprisingly high levels of access to previous psychological therapy. Further work is needed to better understand how using a primarily Web-based trial design may impact on sample characteristics, and the information they provide.

### The Future for ERPonline

ERPonline offers a cheap and easily accessible option for people who are seeking ongoing support following successful treatment, which is currently unavailable. However, given the high functioning and low relapse rates evident in this study, testing the clinical effectiveness of ERPonline for this population would require very large sample sizes. Alternatively, ERPonline could target people at an earlier stage of treatment, who have had not yet received more expensive face-to-face psychological therapy, and need support to understand their mood swings, consider the pros and cons of medication use, and explore the usefulness of monitoring and managing EWS of relapse. For this group, ERPonline may offer a way to reduce the need for expensive individual therapy. Consistent with participant recommendations and previous research, we also need to consider how best to integrate support mechanisms to facilitate use of the intervention, either by integrating the Web-based resource with clinician delivered relapse prevention, or through online peer support as described in other Web-based interventions for BD [10,11,13]. This study highlights the importance of the relationship that the users have with Web-based interventions and how this develops as an extension of the relationship with the humans perceived as offering and supporting its use. Web-based interventions offered in isolation in this context seem unlikely to engage people in the same way and may be perceived negatively as attempts to save money rather than improve care. Our study has explored the feasibility and acceptability of a specific Web-based intervention (ERPonline), but does not address the broader social issue of how acceptable the increasing use of digital health technology is to people with mental health problems [40].

### Strengths of Study

Extensive user involvement improved the content of the ERPonline website, identified recruitment sources, and ensured the measures were appropriate and not too burdensome. The sample was sufficiently large to be able to comment on patterns in the data likely to be indicative of effects on process and outcome measures in a larger trial. Independent randomization, trained blind assessors, and the use of well-established outcome and process measures ensured that the data are reliable and valid. Extensive reflection and learning around feasibility was built into the design process using face-to-face meetings and an online reflection log.

### Limitations

Despite 280 unique site registrations, only 145 people consented, and due to ineligibility and drop out, only 96 were randomized. We have no data on why nearly half the sample registering an interest, then chose not to take part, though for some it may have been delay between prestart expression of interest and randomization. During the trial, we had higher dropout in the ERPonline arm, which is common in trials with a WL control arm and is likely due to the perceived reward of the intervention retaining people through WL. Survey responses were incomplete for feedback on trial participation (22/49 in WL group, 45%) and for feedback on the ERPonline intervention (17/47 in ERPonline arm, 36%). The bias in responders is likely to skew the nature of the feedback which on the whole was very positive.

In summary, we were able to successfully adapt and deliver online a relapse prevention intervention for BD previously used face-to-face. The intervention was successfully evaluated against a WL control group using a RCT design with high levels of retention and data completeness over 48 weeks. Participants had high rates of previous bipolar episodes but had accessed previous psychosocial interventions (where specified, most commonly described as CBT) for BD. Web-based interventions may prove an important cheap, feasible, and acceptable step forward in creating a choice of evidence-based interventions for people with BD at different stages of recovery, but may be more appropriately designed with built-in support and targeted at those with less prior experience of effective care.

## Abbreviations

BBC: British Broadcasting Corporation
BD: bipolar disorder
BIPQ: Brief Illness Perception Questionnaire
BRQ: Bipolar Recovery Questionnair
CBT: cognitive behavior therapy
CSE: Certificate of Secondary Education
CSRI: Client Service Receipt Inventory
CTU: clinical trials unit
DSM-IV: Diagnostic and Statistical Manual of Mental Disorders-IV
ERP: enhanced relapse prevention
EWS: early warning signs
GCSE: General Certificate of Secondary Education
HAM-D: Hamilton Depression Rating Scale
HR: hazard ratio
MARS: Medication Adherence Rating Scale
MRS: Mania Rating Scale
MSIF: Multidimensional Scale of Independent Functioning
NHS: National Health Service
NICE: National Institute for Health and Care Excellence
PG: postgraduate
PSP: Personal and Social Performance Scale
QoLBD: Quality of Life in Bipolar Disorder
RA: Research Associate
RCT: randomized controlled trial
SAE: severe adverse events
SCID: Structured Clinical Interview for Diagnostic and Statistical Manual of Mental Disorders-IV (DSM-IV)
TSC: Trial Steering Committee
WL: waitlist
WSAS: Work and Social Adjustment Scale
